# The association of ferritin with cardiovascular and all-cause mortality in community-dwellers: The English longitudinal study of ageing

**DOI:** 10.1371/journal.pone.0178994

**Published:** 2017-06-07

**Authors:** Nikolaos P. E. Kadoglou, Jane P. Biddulph, Snorri B. Rafnsson, Marialena Trivella, Petros Nihoyannopoulos, Panayotes Demakakos

**Affiliations:** 1 Department of Cardiology, Catharina Hospital, Eindhoven, The Netherlands; 2 Centre for Statistics in Medicine, BOTNAR Research Centre, University of Oxford, Oxford, United Kingdom; 3 Research Department of Epidemiology and Public Health, University College London, United Kingdom; 4 Hammersmith Hospital, Imperial College, London, United Kingdom; Hospital Universitario de la Princesa, SPAIN

## Abstract

**Background:**

Ferritin constitutes a sensitive iron-storage index and multi-functional protein. Evidence on its association with mortality in general population is scarce and conflicting. We investigated the sex-specific associations of ferritin levels with all-cause and cardiovascular mortality in a population-based cohort.

**Methods:**

Data came from the English Longitudinal Study of Ageing and the national mortality registry. The sample comprised 5,471 participants aged ≥52 years. Blood concentration of ferritin was measured at baseline in 2004–05. Sex-specific Cox proportional hazards models were estimated with adjustment for age, major chronic diseases, marital status, educational attainment, total net household wealth, anemia, inflammatory markers, body mass index, smoking, and physical activity. Stratified analyses by chronic disease status were also performed.

**Results:**

We categorized ferritin in sex-specific quartiles. In men, we used, the following categorization: lowest (2-69ng/ml), second lowest (70-118ng/ml), second highest (reference category) (119-193ng/ml) and highest (194-598ng/ml) ferritin quartiles. In women, ferritin was categorized as follows: lowest (2-44ng/ml), second lowest (45-73ng/ml), second highest (reference category) (74-115ng/ml) and highest (116-341ng/ml) ferritin quartiles. 841 deaths of which 262 cardiovascular disease-related were recorded over a mean follow-up time of 7.7 years. Risk for all-cause mortality was found increased in men with hyperferritinemia (194-598ng/ml) and no history of major chronic diseases compared with the reference group [fully-adjusted HR: 1.49 (95%CI 1.03–2.16)]. Among women, those in the lowest ferritin quartile (2-44ng/ml) had increased risk for all-cause mortality [fully-adjusted HR: 1.59 (95%CI 1.18–2.13)] compared with the reference group after adjustment for all covariates. Regarding cardiovascular mortality, we observed a positive association with ferritin levels in men, which was blunted after adjustment for inflammatory markers and lifestyle parameters. Men with no major chronic diseases who were in the highest ferritin quartile had a significantly increased risk of cardiovascular mortality. No association between ferritin levels and cardiovascular mortality was detected in women.

**Conclusion:**

Circulating ferritin levels showed sex-specific prognostic patterns. High ferritin levels in men with no major chronic disease and low ferritin levels in all women were associated with increased all-cause mortality after adjusting for covariates. High ferritin levels in men with no major chronic diseases were also independently associated with an increased risk of cardiovascular mortality. Future research is needed to clarify the prognostic role of ferritin.

## Introduction

Ferritin is an iron-containing protein and its measurement in serum may reliably reflect the human iron storage homeostasis which is essential in fundamental metabolic processes in living organisms [1]. Excessively elevated ferritin concentrations (hyper-ferritinemia) may indicate iron overload, which is toxic for several organs (e.g. myocardium, liver etc) and has been associated with increased morbidity and mortality [2]. On the other hand, low ferritin levels (hypo-ferritinemia) mirrors iron depletion, which is also related to higher morbidity and mortality [3,4].

Ferritin, beyond its function as an iron-storage marker, is a multi-functional protein with possible roles not only in iron delivery, but in proliferation, angiogenesis, and immunosuppression [5]. It is also an acute-phase protein, whose synthesis is driven by cytokines and its levels increase in inflammatory conditions [6]. There are conflicting results highlighting the pluripotency of ferritin as a biomarker in a wide variety of diseases. Previous observational studies have found a positive relationship between high serum ferritin levels and the development of chronic diseases, like coronary artery disease (CAD) [7], cancer [8], and their adverse progression [9]. Other studies failed to find an association between high ferritin levels and chronic diseases [10] or found inverse relationships where low ferritin levels correlated to the development of CAD [11] and cancer [12]. It remains unknown whether ferritin is a bystander or a causative factor in the development of numerous chronic diseases. Moreover, a clear cut-off value of ferritin related to adverse events is still elusive. Despite the potential importance of ferritin as a prognostic biomarker, only a few observational studies have investigated its association with mortality in community samples [13–15]. Those studies reported either a weak association between low ferritin levels and poor prognosis, or failed to demonstrate any association between ferritin levels and survival.

Scarce data support sex differences in ferritin associations with other diseases [16]. Although most women in reproductive age appear with negative iron balance due to poor diet and menstrual blood loss, the underlying mechanisms of gender differences is ferritin level are not entirely attributed to the said negative balance and presumably other unknown mechanisms remain [17]. Our initial hypothesis was that there are sex-specific associations between serum ferritin levels and the risk of all-cause and cardiovascular mortality. Thereby we aimed to explore those sex-differences in a national sample of older community-dwellers. We further examined the impact of covariates, including anemia and inflammation, on the above associations and whether the observed associations held in a subgroup free of major chronic diseases at baseline.

## Material and methods

### Study population

The English Longitudinal Study of Ageing (ELSA) is an ongoing biennial survey of community-d.welling people aged 50 years and older in England, and was designed to be nationally representative. The ELSA sample was drawn from households that had participated in the Health Survey for England (HSE) in 1998, 1999, and 2001 [18]. At wave 1, in 2002–2003, the ELSA sample comprised 11,391 core participants. The first follow-up interview and first health examination took place at wave 2 in 2004–2005, at the respondents’ home. The health examination took place after the interview and involved the collection of blood samples and the measurement of clinical and other health data including anthropometric data. A detailed description of the study can be found at: http://www.elsa-project.ac.uk/. Our study complied with the Declaration of Helsinki, and the locally appointed ethics committee approved the research protocol. An informed consent was obtained from the participants.

For the needs of our analyses, we used interview and health examination data from ELSA wave 2 (2004–2005). Of the 8,780 individuals who participated in wave 2, 6,652 agreed to participate in the health examination, consented and were eventually eligible to give blood samples. Individuals fulfilling any of the following criteria were not eligible for blood sampling: clotting or bleeding disorders, anti-coagulant medication (such as warfarin or acenocoumarol) at the time of interview, history of fits or convulsions, current or recent iron replacement therapy. Of the 6,652 consenting participants, we had valid ferritin measurement for 5,906 participants. The analytical sample comprised 5,471 individuals aged 50 years and older after the exclusion of 373 individuals with missing covariate data or who did not consent to data linkage to the national register of deaths. We also excluded 62 individuals with excessively high ferritin values (i.e. those in the top 1% of the ferritin distribution—30 men with ferritin levels ≥ 599ng/ml and 32 women with ferritin levels ≥ 342ng/ml), as it was likely that they presented with overt pathology.

### Ferritin measurement

After blood collection, samples were dispatched to the Royal Victory Infirmary in Newcastle-upon-Tyne, UK for processing and analysis. Blood samples received by the laboratory later than five days after collection were not used. The concentration of ferritin was measured using Advia Centaur ferritin immunoassay by Bayer. More information on the measurement of ferritin and other blood analytes used in our analysis can be obtained from the HSE 2005 technical report (http://bit.ly/1QvuNwW); both ELSA and the HSE used the same infrastructure and protocols to analyse the blood samples.

There are no established ferritin cut-points to define iron depletion or overload, and therefore, we categorized ferritin into sex-specific quartiles. The distributions of quartiles were as follows: 1) In men: lowest concentration quartile: 2 to 69ng/ml, second lowest concentration quartile: 70 to 118ng/ml, second highest concentration quartile: 119 to 193ng/ml, and highest concentration quartile: 194 to 598ng/ml. 2) In women: lowest concentration quartile: 2 to 44ng/ml, second lowest concentration quartile: 45 to 73ng/ml, second highest concentration quartile: 74 to 115ng/ml, and highest concentration quartile: 116 to 341ng/ml. For statistical analysis purposes, we selected the second highest quartile as the reference category since it showed the lowest relative all-cause and cardiovascular mortality in both men and women.

### Outcome variable

Death registrations up to February 2013 were obtained from the Office for National Statistics for participants consenting to data linkage. Underlying cause of death was classified according to ICD10. Deaths with ICD10 codes I00 to I99 were classified as cardiovascular deaths.

### Covariates

Age, marital status (married versus not), major self-reported doctor diagnosed diseases i.e. heart disease, stroke, cancer, chronic lung disease, and diabetes mellitus, educational attainment (measured using three levels ranging from no qualifications to A-levels or higher), and total net household wealth (divided into tertiles) were considered as potential confounders. Baseline body mass index (<25 kg/m^2^, 25 to <30 kg/m^2^, and ≥30 kg/m^2^), self-reported smoking (current, former, or never smoker), and physical activity (categorized in four categories ranging from no physical activity to vigorous physical activity at least once a week) were included as covariates. Due to the small number of individuals in the underweight category (i.e. BMI<18.5) (n = 28 women and n = 15 men) we merged this category with the normal weight. Anemia was measured using hemoglobin according to the World Health Organization criteria (WHO): <13g/dL in men and <12g/dL in women [19]. High sensitivity C-reactive protein (hsCRP) and fibrinogen were used as markers of inflammation; hsCRP was log transformed because of its markedly skewed distribution. Marital status, socioeconomic position markers such as education and wealth are major determinants of human health and could well be confounding the observed associations.

### Statistical analyses

Multivariable sex-specific Cox proportional hazard regression models were estimated to investigate the associations between ferritin quartiles, cardiovascular and all-cause mortality. We confirmed that the proportionality assumption held using the Schoenfeld residuals test and log-log survival plots.

We sequentially adjusted our models for age (Model 1), then additionally for marital status, educational attainment, total net household wealth, and baseline self-reported doctor diagnosed diseases i.e. heart disease, stroke, cancer, chronic lung disease, and diabetes mellitus (Model 2), anemia (Model 3), hsCRP and fibrinogen (Model 4), and finally body mass index, smoking, and physical activity (Model 5). The adjustments for anemia and inflammation aimed to explore the role of these conditions in the observed associations.

To investigate whether ferritin could be a potential biomarker of future adverse outcomes and mortality amongst older individuals without major chronic diseases at baseline, we estimated models that excluded individuals with the following self-reported doctor-diagnosed chronic diseases: heart disease, stroke, cancer, chronic lung disease, and diabetes. This exclusion might also reduce the possibility of reverse causation i.e. low or high levels of ferritin being a product of baseline health conditions and chronological proximity to death. To further address this issue, in the supplementary analyses, we also excluded deaths that occurred within the first 24 months after the baseline measurements in 2004–2005. In a final step, we excluded individuals with anemia or high levels of hsCRP (i.e. >3mg/L) to determine whether ferritin predicted mortality among people without anemia or chronic low-grade inflammation. All analyses were performed using Stata 13, with a significance threshold of less than 0.05.

## Results

Significant differences in age, socioeconomic position, physical activity, body mass index, anemia, and hsCRP levels by ferritin quartiles were observed in both men and women (Table 1). In the whole population study, serum ferritin concentration was higher in men than in women, with a median of 119ng/ml (IQR: 69-193ng/ml), and 74ng/ml (IQR 44-115ng/ml), respectively. The sensitivity analysis performed was essential to rule out the random effect of baseline clinical conditions, leading to early death (S1 Table).

**Table 1:** Baseline characteristics of men and women aged 50 years and over by ferritin level.

	MEN (n = 2509)					WOMEN (n = 2954)				
	Ferritin quartile					Ferritin quartile				
Model	Lowest	Second lowest	Second highest	Highest		Lowest	Second lowest	Second highest	Highest	
	2-69ng/ml	70-118ng/ml	119-193ng/ml	194-598ng/ml	p-value	2-44ng/ml	45-73ng/ml	74-115ng/ml	116-341ng/ml	p-value
**No. of participants**	640	604	638	627		737	735	740	742	
**Mean (SD) age (years)**					<0.001					0.009
	67.2 (9.9)	65.3 (8.9)	65.5 (9.0)	65.0 (8.8)		66.4 (10.5)	65.9 (9.7)	65.4 (9.4)	67.1 (9.1)	
**Marital status (%)**					0.42					0.13
Married	477 (74.5)	472 (78.2)	486 (76.2)	487 (77.7)		421 (57.1)	452 (61.5)	449 (60.7)	467 (62.9)	
Other	163 (25.5)	132 (21.9)	152 (23.8)	140 (22.3)		316 (42.9)	283 (38.5)	291 (39.3)	275 (37.1)	
**Smoking (%)**					0.53					0.25
Current smoker	80 (12.5)	95 (15.7)	88 (13.8)	98 (15.6)		86 (11.7)	111 (15.1)	118 (15.9)	90 (12.1)	
Former smoker	370 (57.8)	341 (56.5)	380 (59.6)	357 (57.0)		300 (40.7)	294 (40.0)	299 (40.4)	333 (44.9)	
Never smoker	190 (29.7)	168 (27.8)	170 (26.6)	172 (27.4)		351 (47.6)	330 (44.9)	323 (43.7)	319 (43.0)	
**Physical activity (%)**					0.022					0.065
Vigorous physical activity at least once a week	193 (30.2)	226 (37.4)	222 (34.8)	218 (34.8)		186 (25.2)	204 (27.7)	208 (28.1)	176 (23.7)	
Moderate physical activity at least once a week	316 (49.4)	287 (47.5)	318 (49.8)	294 (46.9)		385 (52.2)	349 (47.5)	390 (52.7)	379 (51.1)	
Mild physical activity at least once a week	76 (11.9)	57 (9.4)	60 (9.4)	68 (10.8)		128 (17.4)	133 (18.1)	112 (15.1)	140 (18.9)	
No physical activity at least once a week	55 (8.6)	34 (5.6)	38 (6.0)	47 (7.5)		38 (5.2)	49 (6.7)	30 (4.1)	47 (6.3)	
**Body mass index (%)**					<0.001^c^					<0.001^c^
<25kg/m^2^	188 (29.4)	169 (28.0)	121 (18.9)	115 (18.3)		258 (35.1)	220 (29.9)	234 (31.6)	174 (23.5)	
25 to <30 kg/m^2^	296 (46.3)	290 (48.0)	331 (51.9)	279 (44.5)		254 (34.4)	293 (39.8)	277 (37.4)	277 (37.3)	
≥ 30 kg/m2	126 (19.7)	125 (20.7)	162 (25.4)	201 (32.1)		192 (26.1)	192 (26.2)	202 (27.3)	247 (33.3)	
Missing	30 (4.7)	20 (3.3)	24 (3.8)	32 (5.1)		33 (4.5)	30 (4.1)	27 (3.7)	44 (5.9)	
**Education (%)**					0.036					0.042
A-level or higher	228 (35.6)	245 (40.6)	287 (45.0)	248 (39.5)		177 (24.0)	217 (29.5)	202 (27.3)	169 (22.8)	
GCSE/O-level/other qualification	193 (30.2)	179 (29.6)	163 (25.5)	193 (30.8)		241 (32.7)	247 (33.6)	228 (30.8)	272 (36.7)	
No qualifications	219 (34.2)	180 (29.8)	188 (29.5)	186 (29.7)		319 (43.3)	271 (36.9)	310 (41.9)	301 (40.6)	
**Wealth**					0.003					0.001
Highest tertile (%)	181 (28.3)	216 (35.8)	242 (37.9)	240 (38.3)		205 (27.8)	229 (31.2)	262 (35.4)	248 (33.4)	
Middle tertile (%)	235 (36.7)	213 (35.3)	210 (32.9)	196 (31.3)		233 (31.6)	251 (34.1)	248 (33.5)	246 (33.2)	
Lowest tertile (%)	224 (35.0)	175 (29.0)	186 (29.2)	191 (30.5)		299 (40.6)	255 (34.7)	230 (31.1)	248 (33.4)	
**Anaemia**^a^ **(WHO classification?)**					<0.001					<0.001
Anaemic	76 (11.9)	14 (2.3)	21 (3.3)	25 (4.0)		97 (13.2)	22 (3.0)	28 (3.8)	26 (3.5)	
Non-anaemic	564 (88.1)	590 (97.7)	617 (96.7)	602 (96.0)		640 (86.8)	713 (97.0)	712 (96.2)	716 (96.5)	
**Mean (SD) hsCRP**^b^ **mg/L**					0.003					<0.001
	3.8 (6.8)	3.2 (5.3)	4.2 (7.7)	5.1 (13.8)		3.5 (6.0)	3.5 (5.2)	4.1 (7.1)	5.3 (12.0)	
**Mean (SD) fibrinogen g/L**					0.64					0.073
	3.2 (0.8)	3.2 (0.7)	3.2 (0.8)	3.1 (0.8)		3.3 (0.7)	3.3 (0.7)	3.3 (0.7)	3.3 (0.8)	
**Heart disease**					0.54					0.31
Yes	51 (8.0)	60 (9.9)	51 (8.0)	51 (8.1)		34 (4.6)	28 (3.8)	22 (3.0)	23 (3.1)	
No	589 (92.0)	544 (90.1)	587 (92.0)	576 (91.9)		703 (95.4)	707 (96.2)	718 (97.0)	719 (96.9)	
**Stroke**					0.43					0.20
Yes	31 (4.8)	26 (4.3)	20 (3.1)	23 (3.7)		32 (4.3)	28 (3.8)	20 (2.7)	20 (2.7)	
No	609 (95.2)	578 (95.7)	618 (96.9)	604 (96.3)		705 (95.7)	707 (96.2)	720 (97.3)	722 (97.3)	
**Cancer**					0.76					0.95
Yes	41 (6.4)	35 (5.8)	40 (6.3)	32 (5.1)		57 (7.7)	58 (7.9)	61 (8.2)	63 (8.5)	
No	599 (93.6)	569 (94.2)	598 (93.7)	595 (94.9)		680 (92.3)	677 (92.1)	679 (91.8)	679 (91.5)	
**Chronic lung disease**					0.028					0.45
Yes	63 (9.8)	42 (7.0)	35 (5.5)	49 (7.8)		48 (6.5)	58 (7.9)	43 (5.8)	49 (6.6)	
No	577 (90.2)	562 (93.0)	603 (94.5)	578 (92.2)		688 (93.5)	677 (92.1)	697 (94.2)	693 (93.4)	
**Diabetes Mellitus**					0.009					0.095
Yes	72 (11.3)	44 (7.3)	44 (6.9)	67 (10.7)		55 (7.5)	46 (6.3)	51 (4.5)	23 (6.9)	
No	568 (88.7)	560 (92.7)	594 (93.1)	560 (89.3)		682 (92.5)	689 (93.7)	707 (95.5)	691 (93.1)	

^a^Defined according to the WHO classification of anaemia; In men, haemoglobin concentration <13g/dL, and in women, <12g/dL.

^b^High sensitivity C-reactive protein.

^c^The missing category was not used in the estimation of p-values.

Over a mean follow-up time of 7.7 years, 841 deaths, of which 262 of cardiovascular origin, were recorded in the whole cohort. There was no association between ferritin levels and all-cause mortality in men. However, in the subgroup of men without baseline major chronic disease, high levels of ferritin (i.e. 194-598ng/ml) were associated with an increased risk of mortality independent of all covariates. In women, those with low ferritin levels (2-44ng/ml) were at increased risk of all-cause mortality compared to women in the reference category (second highest ferritin quartile: 74-115ng/ml) irrespective of adjustment for covariates. This association persisted after the exclusion of major baseline chronic diseases (Table 2).

**Table 2:** Association between ferritin and all-cause and cardiovascular mortality by sex.

Model	MEN				WOMEN			
	Ferritin quartile				Ferritin quartile			
	Lowest	Second lowest	Second highest	Highest	Lowest	Second lowest	Second highest	Highest
	2-69ng/ml	70-118ng/ml	119-193ng/ml	194-598ng/ml	2-44ng/ml	45-73ng/ml	74-115ng/ml	116-341ng/ml
**All-cause mortality**								
No. of participants	640	604	638	627	737	735	740	742
No. of deaths	140	106	100	111	122	82	75	100
Person years of follow-up	4718	4582	4869	4732	5619	5756	5824	5758
Model 1 HR (95% CI)^a^	1.19 (0.92–1.53)	1.16 (0.88–1.53)	1.00 (reference)	1.16 (0.89–1.52)	**1.55 (1.16–2.07)**	1.05 (0.77–1.43)	1.00 (reference)	1.22 (0.90–1.65)
Model 2 HR (95% CI)^b^	1.07 (0.83–1.39)	1.11 (0.84–1.45)	1.00 (reference)	1.12 (0.86–1.48)	**1.46 (1.10–1.96)**	1.05 (0.77–1.44)	1.00 (reference)	1.26 (0.93–1.70)
Model 3 HR (95% CI)^c^	1.01 (0.78–1.32)	1.13 (0.86–1.49)	1.00 (reference)	1.12 (0.85–1.47)	**1.44 (1.08–1.93)**	1.06 (0.77–1.45)	1.00 (reference)	1.26 (0.93–1.70)
Model 4 HR (95% CI)^d^	1.04 (0.80–1.36)	1.17 (0.89–1.53)	1.00 (reference)	1.10 (0.84–1.45)	**1.56 (1.17–2.09)**	1.10 (0.80–1.50)	1.00 (reference)	1.25 (0.92–1.69)
Model 5 HR (95% CI)^e^	0.98 (0.75–1.27)	1.16 (0.88–1.53)	1.00 (reference)	1.07 (0.81–1.41)	**1.59 (1.18–2.13)**	1.11 (0.80–1.52)	1.00 (reference)	1.23 (0.91–1.66)
**Excluding participants with chronic diseases**^f^								
No. of participants	433	432	485	446	557	553	580	570
No. of deaths	62	50	54	67	64	51	48	57
Person years of follow-up	3359	3378	3788	3427	4350	4371	4600	4499
Model 1 HR (95% CI)^a^	1.13 (0.79–1.63)	1.16 (0.79–1.70)	1.00 (reference)	**1.52 (1.06–2.18)**	**1.43 (0.99–2.10)**	1.12 (0.75–1.65)	1.00 (reference)	1.12 (0.76–1.64)
Model 2 HR (95% CI)^g^	1.04 (0.72–1.50)	1.09 (0.74–1.60)	1.00 (reference)	**1.54 (1.08–2.21)**	**1.42 (0.98–2.07)**	1.15 (0.78–1.71)	1.00 (reference)	1.19 (0.80–1.75)
Model 3 HR (95% CI)^c^	0.98 (0.67–1.42)	1.10 (0.75–1.62)	1.00 (reference)	**1.56 (1.09–2.24)**	**1.42 (0.97–2.07)**	1.15 (0.78–1.71)	1.00 (reference)	1.18 (0.80–1.74)
Model 4 HR (95% CI)^d^	1.05 (0.72–1.53)	1.16 (0.79–1.71)	1.00 (reference)	**1.60 (1.12–2.30)**	**1.50 (1.03–2.19)**	1.15 (0.77–1.71)	1.00 (reference)	1.16 (0.79–1.71)
Model 5 HR (95% CI)^e^	1.07 (0.73–1.57)	1.16 (0.79–1.72)	1.00 (reference)	**1.49 (1.03–2.16)**	**1.58 (1.08–2.31)**	1.11 (0.75–1.66)	1.00 (reference)	1.12 (0.76–1.66)
**Cardiovascular mortality**								
No. of participants	640	604	638	627	737	735	740	742
No. of deaths	40	31	21	36	42	30	27	33
Person years of follow-up	4718	4582	4869	4732	5619	5756	5824	5758
Model 1 HR (95% CI)^a^	1.53 (0.90–2.60)	1.62 (0.93–2.81)	1.00 (reference)	**1.77 (1.03–3.04)**	1.47 (0.90–2.38)	1.07 (0.64–1.80)	1.00 (reference)	1.14 (0.68–1.89)
Model 2 HR (95% CI)^b^	1.41 (0.82–2.40)	1.50 (0.86–2.61)	1.00 (reference)	**1.79 (1.04–3.07)**	1.43 (0.88–2.33)	1.12 (0.66–1.89)	1.00 (reference)	1.19 (0.71–1.98)
Model 3 HR (95% CI)^c^	1.27 (0.74–2.18)	1.62 (0.93–2.83)	1.00 (reference)	**1.77 (1.03–3.04)**	1.45 (0.89–2.37)	1.11 (0.65–1.88)	1.00 (reference)	1.18 (0.71–1.98)
Model 4 HR (95% CI)^d^	1.35 (0.78–2.31)	1.66 (0.95–2.90)	1.00 (reference)	1.67 (0.97–2.89)	1.60 (0.98–2.62)	1.15 (0.68–1.95)	1.00 (reference)	1.19 (0.71–2.00)
Model 5 HR (95% CI)^e^	1.28 (0.74–2.21)	**1.77 (1.01–3.11)**	1.00 (reference)	1.62 (0.93–2.82)	1.57 (0.95–2.57)	1.09 (0.64–1.86)	1.00 (reference)	1.11 (0.65–1.87)
**Excluding participants with chronic diseases**^f^								
No. of participants	433	432	485	446	557	553	580	570
No. of deaths	17	13	12	18	21	17	17	22
Person years of follow-up	3359	3378	3788	3427	4350	4371	4600	4499
Model 1 HR (95% CI)^a^	1.32 (0.63–2.76)	1.39 (0.64–3.06)	1.00 (reference)	1.83 (0.88–3.80)	1.36 (0.72–2.58)	1.08 (0.55–2.12)	1.00 (reference)	1.27 (0.67–2.40)
Model 2 HR (95% CI)^g^	1.07 (0.51–2.26)	1.17 (0.53–2.58)	1.00 (reference)	2.00 (0.96–4.17)	1.41 (0.74–2.69)	1.12 (0.57–2.20)	1.00 (reference)	1.37 (0.72–2.61)
Model 3 HR (95% CI)^c^	0.97 (0.46–2.08)	1.27 (0.57–2.82)	1.00 (reference)	**2.15 (1.02–4.50)**	1.41 (0.74–2.70)	1.10 (0.56–2.17)	1.00 (reference)	1.37 (0.72–2.61)
Model 4 HR (95% CI)^d^	1.11 (0.52–2.38)	1.38 (0.62–3.08)	1.00 (reference)	**2.23 (1.06–4.69)**	1.52 (0.79–2.91)	1.09 (0.55–2.15)	1.00 (reference)	1.35 (0.71–2.58)
Model 5 HR (95% CI)^e^	1.23 (0.56–2.70)	1.56 (0.69–3.51)	1.00 (reference)	**2.24 (1.03–4.87)**	1.55 (0.80–2.98)	0.97 (0.48–1.93)	1.00 (reference)	1.20 (0.62–2.30)

HR = Hazard ratio; CI = Confidence interval; Significant results in bold

^**a**^Adjusted for age.

^**b**^As model 1, plus adjustment for marital status, education, wealth, and baseline self-reported doctor diagnosed diseases i.e. heart disease, stroke, cancer, chronic lung disease, and diabetes mellitus.

^c^As model 2, plus adjustment for anaemia defined according to the WHO classification of anaemia; In men, haemoglobin concentration <13g/dL, and in women, <12g/dL.

^d^As model 3, plus adjustment for log high sensitivity C-reactive protein and fibrinogen.

^e^As model 4, plus adjustment for smoking, physical activity and body mass index.

^f^ Self-reported doctor diagnosis of heart disease, stroke, cancer, chronic lung disease, and diabetes mellitus.

^g^As model 1, plus adjustment for marital status, education, and wealth.

In men, there was a positive association between highest ferritin quartiles and the risk of cardiovascular mortality after adjustment for age, chronic diseases and anemia. Adjustment for additional covariates (lifestyle, inflammation) attenuated this association and in this case men with ferritin levels belonging to the second lowest quartile showed significantly increased cardiovascular mortality than those in the reference category (i.e. second highest ferritin quartile: 119-193ng/ml) (Table 2). Among men without major baseline chronic diseases, those with the highest ferritin levels had considerably increased risk of cardiovascular mortality compared to reference group after full adjustment for covariates (models 3, 4 and 5). Women with low ferritin levels (2-44ng/ml) were at increased risk of cardiovascular mortality, although this association did not reach statistical significance.

## Discussion

The present analysis was based on a national sample of community-dwelling people aged 50 years or older and used one of the largest known samples (ELSA) to explore the sex-specific prognostic role of ferritin. Healthy men with hyperferritenemia (194-598ng/ml) were at a significantly increased risk of all-cause and cardiovascular mortality compared to the reference category. In the entire male population, the increased risk for cardiovascular mortality hyperferritinemia was attenuated after adjustment for inflammatory and lifestyle covariates. Moreover, our findings indicated significantly higher hazard for all-cause mortality amongst women with low ferritin serum levels (2-44ng/ml), which persisted after adjustment for covariates.

A growing body of evidence has suggested that excessive values of ferritin, either low or high, may be predictive of increased mortality in patients with hematological [20], kidney, metabolic, cardiovascular [21], neoplasmatic [22], and other diseases. Regarding all-cause mortality in men, we found a positive association with high ferritin quartiles, but only in the subgroup without any baseline major chronic disease. Because of this variation by chronic disease status, we hypothesized that the association between high ferritin levels and all-cause mortality in the general male population may be obscured by comorbidities. Other population studies have provided weak evidence of such association among people without medical history of chronic diseases [23]. Further research on the prognostic role of ferritin amongst healthy men is required.

In men, most studies have shown either positive or no relationship between circulating ferritin and cardiovascular mortality independent of other causative factors [24,25]. Conversely, a single study has documented low ferritin in 196 patients with a first non-ST elevation acute coronary syndrome and high mortality risk [26]. To our knowledge, this is the first study demonstrating a significant relationship between high ferritin levels and cardiovascular mortality in relatively healthy men with no major chronic diseases. However, the addition of the full range of potential confounding factors, as well as markers of inflammation and lifestyle (like smoking, physical activity and obesity) blunted this association in the male group, indicating their higher influence over other factors. Nevertheless, the overall interaction between ferritin levels and cardiovascular mortality seems to be more complex. Men without pre-existent chronic diseases and high ferritin levels (194-598ng/ml) appeared with increased cardiovascular death risk independent of confounders. This has a clinical implication, since measured ferritin levels higher from reference spectrum may add significant value in cardiovascular prognosis in otherwise healthy men. A mechanistic explanation of all the aforementioned interactions cannot be provided in the framework of an observational study. A recent, large, general population survey demonstrated a U-shaped relationship of another marker of iron status—transferrin saturation ratio (serum iron/total iron binding capacity)—with cardiovascular mortality [27].

Evidence on the association between ferritin and mortality in women is limited. A recent study of a general population sample indicated increased ferritin concentrations (>200ng/ml) as an independent predictor of premature death, with the association following a linear relationship [27]. Other studies reported a U-shaped association among women, between ferritin and cardiovascular mortality [14] and morbidity [28]. In our study, women in the lowest quartile of ferritin levels had higher all-cause and cardiovascular mortality risk compared to those in the reference quartile, albeit the latter did not reach significance. These associations remained unchanged after adjustment for covariates including anemia and inflammatory markers. Subgroup analysis also confirmed poor prognosis in women with low serum ferritin in the absence of baseline chronic diseases. It should be noted that women in the lower ferritin quartile do not necessarily suffer from iron stores depletion using the WHO definition [29]. Thus, our female population with otherwise “low normal” iron store and without anemia had an elevated risk for premature death, which is of clinical importance.

The mechanism underlying the sex-specific associations between circulating ferritin and mortality is likely to be complex, and may not simply reflect changes in body iron stores with apparent clinical consequences [4]. For this reason, we excluded patients with excessive hyperferritinemia indicating hemochromatosis, while anemia entered our adjustment models to ascertain that it is not iron deficiency that drives the results. The most important finding of this study was the sex-related relationship between ferritin and mortality. Although sex differences in the distribution of ferritin levels have previously been reported [30], it is still unknown which iron-dependent or iron-independent mechanisms differentiate the association between ferritin and mortality in men and women. Presumably, males and females respond differently to iron-induced myocardial injury, as this has been implicated in an experimental study [31]. In parallel, elevated ferritin concentrations in men have been significantly related to higher risk of metabolic diseases (for instance, hyperlipidemia, obesity, and diabetes) with consequent increase of cardiovascular risk [16]. Notably, those relationships were not confirmed among women. This gives rise to the assumption of sex-dependent differences in the expression of regulatory molecules involved in iron metabolism or the non-iron related processes of ferritin [32]. Nevertheless, the explanation of this sex-dependent behavior of ferritin is an unwieldy process. Moreover, applying sex-specific normal range of ferritin concentrations may be considered for clinical decisions in the future.

Besides this, inflammatory processes may influence circulating ferritin levels. In the context of cardiovascular disease, ferritin may exert a dual pro-inflammatory role. Its increased levels may represent an acute phase reactant equivalently to hsCRP [33], while the reduced concentration may also precipitate inflammation [34]. In summary, both high and low ferritin levels may relate to enhanced inflammation, inducing the progression of cardiovascular and other chronic diseases. In comparison to previous studies, the strength of our analysis was that it took into consideration the impact of inflammation on ferritin levels. After adjustment for well-established and sensitive markers of inflammation (CRP and fibrinogen), results remained unaffected, which may outline the multi-functional role of ferritin, potentially involved in other non-inflammatory pathways (e.g. angiogenesis or proliferation).

The present work has some limitations. First, our data lacked information on the use of medication which is necessary for a mechanistic interpretation of the observed associations; this might in part explain the observed variation of the association between ferritin and all-cause mortality in men by chronic disease. Second, although we carried out sensitivity analyses to limit the potential confounding effect of major chronic diseases, we cannot ignore the possibility of unaccounted confounders having influenced our results. Third, in the absence of widely accepted and validated ferritin cut-off points, we used sex-specific quartiles as an acceptable and unbiased alternative categorization. However, their clinical relevance and discriminatory power in the general population or groups with certain diseases remains unknown. In parallel, all blood samples were momentarily measured at baseline and may have been affected by transient factors. Although this is a common research practice we cannot rule out the impact of unknown factors on the association between a single, snapshot measurement, and long-term mortality. Lastly, the observational design of our study precludes any causal inferences and therefore our findings should be interpreted with caution.

In conclusion, circulating ferritin levels showed sex-specific prognostic patterns in a community-based cohort. The risks of all-cause and cardiovascular mortality were increased among healthy men with hyperferritinemia. Considering the whole male population, the highest ferritin quartile showed an association to cardiovascular mortality, but this association was blunted after adjustment for inflammation and lifestyle covariates. Women with low serum ferritin levels appeared to have higher all-cause mortality risk after adjustment for numerous covariates. Therefore, ferritin levels may not only reflect iron store status, but also be of paramount importance in risk stratification of community-dwellers, tailoring pre-emptive therapy. The rapid, sensitive, validated and low-cost blood assay makes ferritin a feasible and widely used biomarker, and its potential implications for public health warrant further investigation.

## Supporting information

S1 Table The associations between ferritin and mortality after excluding participants who died within the first 24 months since the baseline interview, and participants with anaemia or increased of hsCRP levels. (DOCX)
