# Supplementary material for: The association of ferritin with cardiovascular and all-cause mortality in community-dwellers: The English longitudinal study of ageing
Source: PLoS One. 2017 Jun 7;12(6):e0178994. doi: 10.1371/journal.pone.0178994 (PMC5462410; doi:10.1371/journal.pone.0178994)
Supplement: S1 Table — (DOCX) [file pone.0178994.s001.docx]

**S1 Table: The associations between ferritin and mortality after excluding participants who died within the first 24 months since the baseline interview, and participants with anaemia or increased of hsCRP levels**

|  |  | **Men** | | |  | **Women** | | | |
| --- | --- | --- | --- | --- | --- | --- | --- | --- | --- |
|  |  | **Ferritin quartile** | | |  | **Ferritin quartile** | | | |
| **Model** | **Lowest** | **Second lowest** | **Second highest** | **Highest** |  | **Lowest** | **Second lowest** | **Second highest** | **Highest** |
|  | **2-69ng/ml** | **70-118ng/ml** | **119-193ng/ml** | **194-598ng/ml** |  | **2-44ng/ml** | **45-73ng/ml** | **74-115ng/ml** | **116-341ng/ml** |
| **All-cause mortality** | | | | | | | | | |
| **Excluding deaths that occurred within the first 24months since the baseline interview** | | | | | | | | | |
| No of participants | 611 | 592 | 620 | 602 |  | 719 | 725 | 729 | 728 |
| No of deaths | 111 | 94 | 82 | 86 |  | 104 | 72 | 64 | 86 |
| Person years of follow-up | 4688 | 4568 | 4845 | 4717 |  | 5599 | 5744 | 5809 | 5746 |
| Fully adjusted HR (95% CI)^a^ | 0.93 (0.69-1.26) | 1.23 (0.91-1.66) | 1.00 (reference) | 0.98 (0.72-1.34) |  | **1.65 (1.20-2.26)** | 1.15 (0.81-1.61) | 1.00 (reference) | 1.24 (0.90-1.73) |
| **Excluding participants with anaemia^b^** | | | | | | | | | |
| No of participants^a^ | 564 | 590 | 617 | 602 |  | 640 | 713 | 712 | 716 |
| No of deaths | 107 | 99 | 84 | 95 |  | 94 | 77 | 67 | 91 |
| Person years of follow-up^a^ | 4229 | 4503 | 4773 | 4589 |  | 4938 | 5599 | 5624 | 5590 |
| Fully adjusted HR (95% CI)^d^ | 1.14 (0.85-1.53) | 1.26 (0.94-1.70) | 1.00 (reference) | 1.21 (0.90-1.63) |  | **1.58 (1.15-2.18)** | 1.07 (0.77-1.50) | 1.00 (reference) | 1.24 (0.90-1.71) |
| **Excluding participants with high C-reactive protein levels^c^** | | | | | | | | | |
| No of participants | 424 | 429 | 412 | 402 |  | 487 | 481 | 455 | 397 |
| No of deaths | 67 | 60 | 52 | 53 |  | 73 | 47 | 39 | 39 |
| Person years of follow-up | 3253 | 3323 | 3221 | 3118 |  | 3748 | 3797 | 3593 | 3128 |
| Fully adjusted HR (95% CI)^a^ | 0.80 (0.55-1.18) | 1.04 (0.71-1.52) | 1.00 (reference) | 0.84 (0.57-1.25) |  | 1.40 (0.94-2.08) | 0.95 (0.61-1.47) | 1.00 (reference) | 0.87 (0.55-1.37) |
| **Cardiovascular mortality** | | | | | | | | | |
| **Excluding deaths that occurred within the first 24months since the baseline interview** | | | | |  |  |  |  |  |
| No of participants | 611 | 592 | 620 | 602 |  | 719 | 725 | 729 | 728 |
| No of deaths | 28 | 27 | 17 | 30 |  | 34 | 26 | 22 | 28 |
| Person years of follow-up | 4688 | 4568 | 4845 | 4717 |  | 5599 | 5744 | 5809 | 5746 |
| Fully adjusted HR (95% CI)^a^ | 1.04 (0.55-1.97) | **1.94 (1.05-3.60)** | 1.00 (reference) | 1.56 (0.84-2.91) |  | 1.64 (0.95-2.85) | 1.23 (0.69-2.21) | 1.00 (reference) | 1.19 (0.67-2.12) |
| **Excluding participants with anaemia^b^** | | | | | | | | | |
| No of participants^a^ | 564 | 590 | 617 | 602 |  | 640 | 713 | 712 | 716 |
| No of deaths | 27 | 27 | 15 | 31 |  | 33 | 27 | 23 | 32 |
| Person years of follow-up^a^ | 4229 | 4503 | 4773 | 4589 |  | 4938 | 5599 | 5624 | 5590 |
| Fully adjusted HR (95% CI)^d^ | 1.63 (0.85-3.12) | **2.12 (1.11-4.07)** | 1.00 (reference) | **2.26 (1.19-4.29)** |  | 1.55 (0.90-2.66) | 1.00 (0.57-1.77) | 1.00 (reference) | 1.18 (0.68-2.05) |
| **Excluding participants with high C-reactive protein levels^c^** | | | | | | | | | |
| No of participants | 424 | 429 | 412 | 402 |  | 487 | 481 | 455 | 397 |
| No of deaths | 10 | 15 | 10 | 15 |  | 73 | 47 | 39 | 39 |
| Person years of follow-up | 3253 | 3323 | 3221 | 3118 |  | 3748 | 3797 | 3593 | 3128 |
| Fully adjusted HR (95% CI)^a^ | 0.47 (0.17-1.24) | 1.63 (0.70-3.79) | 1.00 (reference) | 1.20 (0.51-2.82) |  | 1.59 (0.78-3.22) | 1.18 (0.54-2.59) | 1.00 (reference) | 0.72 (0.31-1.67) |
| ^a^ Adjusted for age, baseline self-reported doctor diagnosed diseases (i.e. heart disease, stroke, cancer, chronic lung disease, and diabetes mellitus), marital status, education, wealth, anaemia, log high sensitivity C-reactive protein, fibrinogen, smoking, physical activity and body mass index.  ^b^ Excluding men with haemoglobin <13g/dl and women with haemoglobin <12g/dl  ^c^ Excluding participants with high sensitivity C-reactive protein ≥3mg/L  ^d^ Adjusted for age, baseline self-reported doctor diagnosed diseases (i.e. heart disease, stroke, cancer, chronic lung disease, and diabetes mellitus), marital status, education, wealth, log high sensitivity C-reactive protein, fibrinogen, smoking, physical activity and body mass index. | | | | | | | | | |
